# Endobronchial optical coherence tomography helps to estimate the cartilage damage of the central airway in TBTB patients

**DOI:** 10.3389/fcimb.2023.1278281

**Published:** 2023-11-30

**Authors:** Kaican Zong, Bin Liu, Shiying Li, Yishi Li, Shuliang Guo

**Affiliations:** ^1^ Department of Respiratory Medicine, The First Affiliated Hospital, Chongqing Medical University, Chongqing, China; ^2^ Department of Respiratory Medicine, The Central Hospital Affiliated Chongqing University of Technology, Chongqing, China; ^3^ Department of Infectious Diseases, Key Laboratory of Molecular Biology for Infectious Diseases (Ministry of Education), Institute for Viral Hepatitis, The Second Affiliated Hospital, Chongqing Medical University, Chongqing, China

**Keywords:** endobronchial optical coherence tomography, tracheobronchial tuberculosis, cartilage, central airway, airway wall

## Abstract

**Purpose:**

At present, there are few examination methods used to evaluate tracheobronchial cartilage damage. In our study, we explored whether endobronchial optical coherence tomography (EB-OCT) can be used to estimate central airway cartilage damage in tracheobronchial tuberculosis (TBTB) patients.

**Methods:**

In our study, we used the OCTICS Imaging system to perform EB-OCT scanning for TBTB patients. The thickness of the central airway wall and cartilage was measured by the OCTICS software system workstation.

**Results:**

There were 102 TBTB patients included in our study cohort. Their EB-OCT images of the central airway cartilage showed that abnormal cartilage manifests as thinning of the cartilage, cartilage damage, cartilage destruction, and even cartilage deficiency. The cartilage morphology becomes irregular and discontinuous. Some parts of the cartilage become brighter in grayscale. The intima of the cartilage is thickened and discontinuous, and the boundary with submucosa and mucosa is unclear.

**Conclusion:**

Our study conducted EB-OCT examination of the central airway cartilage of TBTB patients *in vivo* for the first time. EB-OCT helps to estimate the cartilage damage of the central airway in TBTB patients to some extent.

## Introduction

Tracheobronchial cartilage is an important supporting structure of the airway. A complete airway cartilage ring can maintain normal airway morphology and prevent airway collapse (Turcatel et al., 2013; Snijders and Barbato, 2015). Damage to the cartilage ring can cause deformation of the lumen, resulting in varying degrees of stenosis, and airway stenosis can lead to a series of adverse clinical consequences, such as atelectasis, severe breathing difficulties, and asphyxia (Snowball et al., 2015; Turcatel et al., 2017). However, there are currently few examination methods used to evaluate whether the tracheobronchial cartilage is damaged. Some studies have found that endobronchial optical coherence tomography (EB-OCT) imaging can reveal fine-layered features of the normal bronchial wall, including mucosa, submucosa, and cartilage (Su ZQ. et al., 2019; Goorsenberg et al., 2020; Zhang et al., 2023). EB-OCT can achieve a tissue resolution of 10 μm and maintain a high degree of consistency with tissue pathology (Su ZQ. et al., 2019; Zhang et al., 2023).

As far as we know, there is currently no study that explores whether EB-OCT can be used to evaluate central airway cartilage damage in tracheobronchial tuberculosis (TBTB) patients. As is known to all, TBTB was considered the main cause of benign airway stenosis (Shahzad and Irfan, 2016; Zhuang et al., 2023). TBTB is a special clinical type of tuberculosis. The available data show that approximately 10%–40% of patients with PTB are complicated by TBTB (Lee et al., 2010; Su Z. et al., 2019). With the popularity of bronchoscopy, the incidence of TBTB may actually be up to 54.3% (Jung et al., 2015). The characteristic of TBTB is that it can infect any portion of the tracheobronchial tree and may affect any layer of the tracheobronchial wall (Su Z. et al., 2019). It often results in tracheobronchial stenosis, which can lead to repeated hospitalizations due to breathing difficulties and lung infections. Moreover, more than two-thirds of patients develop severe bronchial stenosis and stricture formation despite adequate medical treatment (Su Z. et al., 2019; Hu et al., 2022). Refractory tracheobronchial stenosis may eventually lead to a decline in pulmonary function, respiratory failure, or death (Hu et al., 2022). Bronchoscopy is the main method to diagnose TBTB, but it cannot detect the fine-layered features of a normal bronchial wall, especially the airway cartilage. At present, some studies have found that EB-OCT can be used to measure the airway wall in many different lung diseases or airway diseases (Zhou et al., 2020; Kalverda et al., 2022). Therefore, this study aims to explore the value of EB-OCT in evaluating central airway cartilage damage in TBTB patients. If EB-OCT is used to evaluate airway cartilage damage in TBTB patients, we will have a more intuitive understanding of the airway cartilage damage in these patients. At the same time, we can also distinguish the degree of airway cartilage damage in different types of TBTB patients through EB-OCT. Next, the cartilage damage situation indicated by EB-OCT may guide us in the selection of airway intervention treatment measures and also allow us to predict the efficacy of different airway intervention measures.

## Patients and methods

In our cohort study, TBTB patients were consecutively admitted to The First Affiliated Hospital of Chongqing Medical University from 4 May 2023 to 31 July 2023. We collected the data of each patient, including their sex, age, number of lesions involved, main site of lesions, main subtype of TBTB, and EB-OCT imaging of the central airway. Based on EB-OCT imaging, the thickness of the airway wall was measured both in the main site of lesions and in the relatively normal central airway in TBTB patients. In our study, the thickness of the airway wall mainly included the thickness from the mucosa to the intima of the cartilage (epithelium, lamina propria, and submucosa) and cartilage (including its inner and outer membranes). The inclusion criterion was that all patients were confirmed with a diagnosis of TBTB according to the *Diagnosis and Treatment Guideline for Tracheobronchial Tuberculosis*, which was published by the Chinese Medical Association (2012). The exclusion criterion was patients less than 18 years old. The study was approved by the Research Ethics Commission of the First Affiliated Hospital of Chongqing Medical University, and the patients’ written informed consent to this study was provided before each operation according to relevant policies.

We used the OCTICS Imaging (Guangzhou Winstar Medical Technology Company Limited, Guangzhou, China) system to perform the EB-OCT scanning. The OCTICS Imaging system can automatically process the collected images, generate clinically recognizable images, and display the hierarchical structure of the airway wall and the condition of the tracheal cartilage. The EB-OCT probe was 1.7 mm in outer diameter with automated rotating and rotary auto-pullback functions. Generally speaking, the operation of OCT is very simple, and it can be operated by performing bronchoscopy. It is similar to operating a small ultrasound probe during bronchoscopy. The OCT probe was placed into the airway through the bronchoscope operation channel, and the target area was scanned. EB-OCT scanning was performed from the distal to the proximal central airway and scanned repeatedly in the area of interest. The thickness of the central airway wall was measured by the OCTICS software system workstation. To measure the thickness of the tracheal wall, we chose the location with the clearest OCT image, especially the one that can display the structure of the cartilage. We selected three measuring points with an average interval of 0.5 mm and took their average values as the thickness of the airway wall.

### Statistical analysis

The statistical analysis was performed with SPSS 26.0. Data were expressed as mean ± standard deviation (SD) for the parameters with a Gaussian distribution. Parametric (Student’s) or non-parametric (Mann–Whitney *U*) tests were used to compare differences. Paired sample *t*-test and independent sample *t*-test were used to compare the airway wall thickness of different groups. If two-sided *p*-values were less than 0.05, we considered them statistically significant.

## Results

### Baseline and clinical characteristics

**Figure 1:**
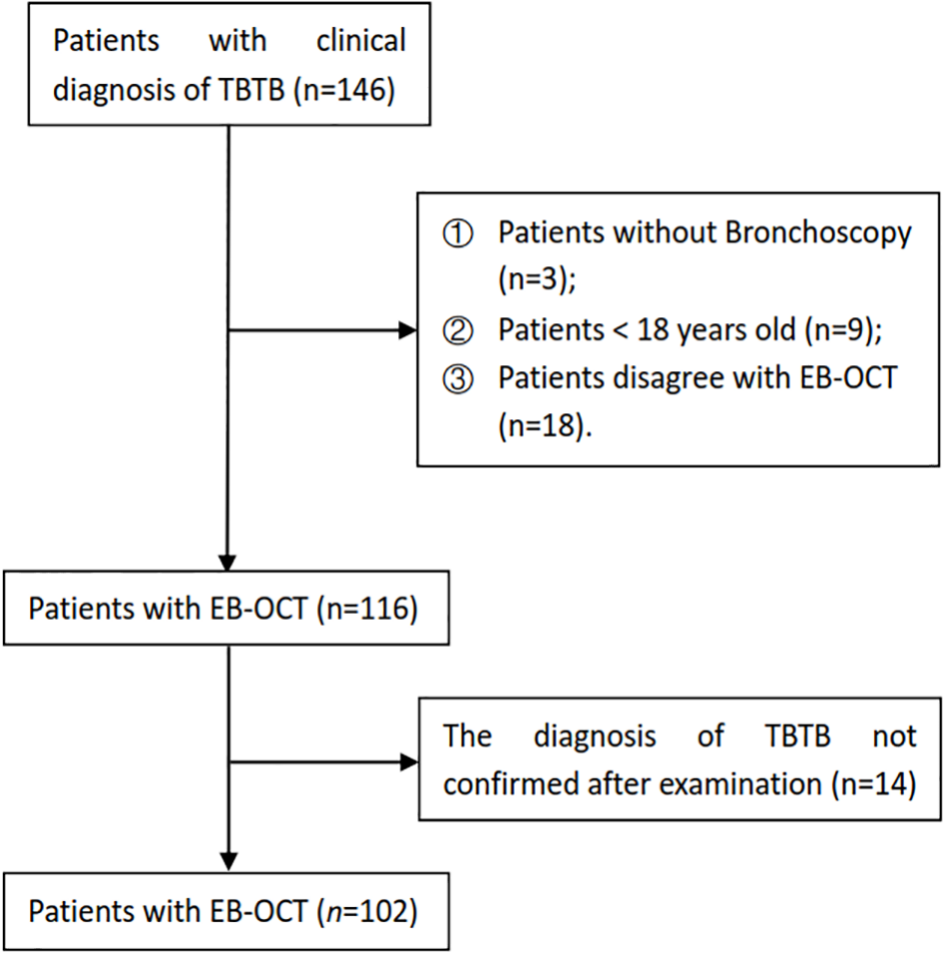
Flowchart of tracheobronchial tuberculosis (TBTB) patient selection.

A total of 146 patients had a clinical diagnosis of TBTB; some patients without bronchoscopy and EB-OCT and who were less than 18 years old were excluded. After examination, some patients whose diagnosis of TBTB was not confirmed were excluded as well. Finally, there were 102 TBTB patients recruited to our study cohort (**Figure 1**). The baseline and clinical characteristics of these patients are shown in **Table 1**. According to the guideline (Chinese Medical Association, 2012), TBTB patients were classified into six types: inflammatory infiltration (II), ulcer necrosis (UN), granulation hyperplasia (GH), cicatricial stenosis (CS), tracheobronchial malacia (TM), and lymph fistula (LF). In our study, we did not analyze the type of lymph fistula because it involved lymph nodes outside the airway. The cicatricial stenosis and tracheobronchial malacia were the main types in our cohort, comprising 89% of all patients (**Table 1**).

**Table 1:** The baseline and clinical characteristics of tracheobronchial tuberculosis patients.

Variable	Values (*n* = 102)
Age, years (mean ± SD)	38.6 ± 14.3
Female	68 (66.7%)
Male	34 (33.3%)
Number of lesions involved	
Single	28 (27.5%)
Multiple	74 (72.5%)
Main site of lesions	
Trachea	11 (10.8%)
Right main bronchus	25 (24.5%)
Right bronchus intermedius	20 (19.6%)
Left main bronchus	46 (45.1%)
Main subtype of TBTB	
Inflammatory infiltration	3 (2.9%)
Ulcer necrosis	2 (2.0%)
Granulation hyperplasia	7 (6.9%)
Cicatricial stenosis	64 (62.7%)
Tracheobronchial malacia	25 (24.5%)
Lymph fistula	1 (0.8%)

TBTB, tracheobronchial tuberculosis; SD, standard deviation.

### EB-OCT image

**Figure 2:**
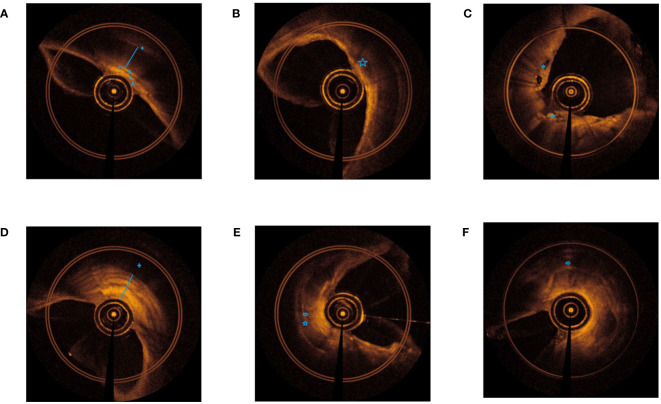
EB-OCT imaging of central airway in different subtypes of TBTB patients and their relatively normal airway. **(A)**. Normal airway: clear boundaries of each layer of the airway wall (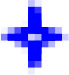 outer membrane of cartilage, 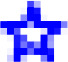 cartilage, 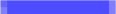 inner membrane of cartilage, 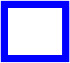 submucosa, 
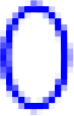
 lamina propria, 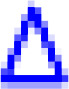 epithelium). **(B)**. Inflammatory infiltration: the hierarchical structure of the airway wall is unclear, with low gray inflammatory exudation changes and bright gray dots, and the morphology of airway cartilage is normal (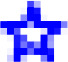 low gray inflammatory exudation). **(C)**. Ulcer necrosis: destruction of mucosa and submucosa, low gray necrotic tissue is visible, and airway cartilage is visible (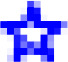 low gray necrotic tissue, 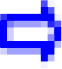 destruction of mucosa and submucosa). **(D)**. Granulation hyperplasia: thickening of the mucosa and submucosa (strong signal reflection band) with narrow lumen, some layers projecting inward, and discontinuity of the cartilage layer (low signal reflection band). The time of airway remodeling varies, and there are visible changes of billowing wheat in the airway wall (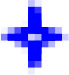 cartilage not fully displayed, 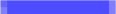 obvious thickening of mucosa and submucosa). **(E)**. Cicatricial stricture: normal structures of mucosa and submucosa disappear, with broad bright signals and narrow lumen, and may be with damaged or destructive cartilage (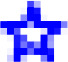 damaged or destructive cartilage, 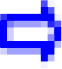 normal structures of mucosa and submucosa disappear). **(F)**. Tracheobronchial malacia: normal structures of mucosa and submucosa disappear, with broad bright signals and obviously narrow lumen, with cartilage destruction, and even cartilage deficiency (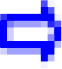 cartilage destruction, and even cartilage deficiency).

To understand the EB-OCT imaging of the central airway wall in TBTB patients, we presented the images of the central airway wall and its tracheal cartilage in different subtypes of TBTB patients in **Figure 2**. From the EB-OCT imaging, we found that a normal airway cartilage is shown as a regular narrow shape, such as an arch bridge. The grayscale of the cartilage is darker, while the grayscale of its inner and outer membranes is brighter. Its inner and outer membranes are intact, uniform, and continuous. Abnormal cartilage manifests as thinning of cartilage, cartilage damage, cartilage destruction, and even cartilage deficiency. The cartilage morphology becomes irregular and discontinuous. Some parts of the cartilage become brighter in grayscale. The intima of the cartilage is thickened and discontinuous, and the boundary with submucosa and mucosa is unclear. These images provided us with an intuitive understanding of the changes of the central airway wall and its tracheal cartilage in TBTB patients. The cartilage had varying degrees of damage in the subtype of cicatricial stricture and tracheobronchial malacia, even cartilage deficiency. However, some of the cartilage in the subtype of granulation hyperplasia was invisible. This may be due to the obvious thickening of mucosa and submucosa in these types of patients, which exceeded the detection range of EB-OCT.

Next, from a sex aspect, we measured the thickness of the airway wall of TBTB patients in their relatively normal central airway based on EB-OCT imaging (**Table 2**). There were no significant differences in thickness from the mucosa to the intima of the cartilage in different areas of the central airway between the male and female participants. The mean thickness from the mucosa to the intima of the cartilage was from 369 μm to 454 μm. It showed no significant difference in the right main bronchial cartilage between the male and female participants, while the mean thickness of the tracheal cartilage, right bronchus intermedius cartilage, and left main bronchial cartilage was significantly different between the male and female participants. The mean thickness of tracheal cartilage in the male participants could reach 1,935 μm, which was the thickest among these patients, while the measurable thickness of EB-OCT could reach 3 mm. This suggests that for most TBTB patients, EB-OCT can clearly display the structure of the tracheal wall, including the cartilage, even in the thickest part of the cartilage.

**Table 2:** The thickness of airway wall of TBTB patients in their relatively normal central airway in different sexes based on EB-OCT imaging.

Thickness	Female (μm) (mean ± SD)	Male (μm) (mean ± SD)	*P*-value
Mucosa to intima of cartilage			
Tracheal (*n* = 20)	419 ± 95	443 ± 51	0.411
Right main bronchus (*n* = 10)	454 ± 74	364 ± 81	0.074
Right bronchus intermedius (*n* = 12)	384 ± 63	409 ± 105	0.458
Left main bronchus (*n* = 21)	369 ± 75	369 ± 72	0.993
Cartilage			
Tracheal (*n* = 20)	1306 ± 136	1935 ± 227	<0.001
Right main bronchus (*n* = 10)	1217 ± 207	1236 ± 119	0.749
Right bronchus intermedius (*n* = 12)	1030 ± 146	878 ± 136	0.008
Left main bronchus (*n* = 21)	1073 ± 168	1249 ± 254	0.041

EB-OCT, endobronchial optical coherence tomography.

Furthermore, from a different location aspect, we compared the thickness of the airway wall of TBTB patients in their relatively normal central airway based on EB-OCT imaging (**Table 3**). The results showed that the mean thickness from the mucosa to the intima of the cartilage in the tracheal cartilage was significantly thicker than that of the left main bronchus. In addition, there was no significant difference in the mean thickness from the mucosa to the intima of the cartilage between the trachea, right main bronchus, right intermediate bronchus, and left main bronchus, while there was a significant difference in the thickness of the airway cartilage between different parts of the central airway. The results showed us the airway wall and cartilage thickness of the trachea, right main bronchus, right intermediate bronchus, and left main bronchus in TBTB patients.

**Table 3:** Comparison of the thickness of the relatively normal central airway wall of TBTB patients in different locations based on EB-OCT imaging.

Thickness	Mucosa to intima of cartilage (μm) (mean ± SD)		*P*-value	Cartilage (μm) (mean ± SD)		*P*-value
Tracheal vs. RMB (*n* = 20)	443 ± 51	409 ± 88	0.079	1,589 ± 369	1,267 ± 164	<0.001
Tracheal vs. RBI (*n* = 24)	426 ± 68	396 ± 85	0.115	1,760 ± 314	954 ± 150	<0.001
Tracheal vs. LMB (*n* = 40)	431 ± 76	368 ± 75	0.001	1,564 ± 372	1,153 ± 242	<0.001
RMB vs. RBI (*n* = 20)	409 ± 88	399 ± 90	0.687	1,217 ± 157	954 ± 150	<0.001
RMB vs. LMB (*n* = 20)	409 ± 88	369 ± 74	0.208	1,267 ± 164	1,133 ± 226	0.001
RBI vs. LMB (*n* = 24)	396 ± 85	383 ± 77	0.577	954 ± 150	1,177 ± 235	<0.001

RMB, right main bronchus; RBI, right bronchus intermedius; LMB, left main bronchus.

We further compared the thickness of the central airway wall in different subtypes of TBTB patients based on EB-OCT imaging (**Table 4**). The results showed that the mean thickness from the mucosa to the intima of the cartilage in the relatively normal group was significantly thinner than that in five subtypes of TBTB patients. The mean thickness from the mucosa to the intima of the cartilage in the granulation hyperplasia subtype was significantly thicker than that in other subtypes. The mean thickness of cartilage in the relatively normal group was significantly thicker than that in ulcer necrosis (1,597 ± 368 vs. 1,474 ± 28, *p* = 0.021) and CICATRICIAL stenosis (1,597 ± 368 vs. 1,217 ± 430, *p* < 0.001). The mean thickness of the cartilage in the inflammatory infiltration subtype was significantly thicker than that in cicatricial stenosis (1,506 ± 59 vs. 1,217 ± 430, *p* = 0.001), while the mean thickness of the cartilage was not significantly different between the relatively normal group and the inflammatory infiltration subtype (1,597 ± 368 vs. 1,506 ± 59, *p* = 0.135). Because some of the cartilage in the granulation hyperplasia subtype was invisible and the cartilage in the tracheobronchial malacia subtype was destroyed or deficient, we were unable to obtain measurement data for cartilage thickness. Thus, the mean thickness of the cartilage in these two subtypes did not compare with that of other subtypes. These results suggested that the cartilage in each of these five subtypes of TBTB patients may be affected and changed, which included the intima of the thicker cartilage, thinner cartilage, cartilage destruction, and even cartilage deficiency.

**Table 4:** Comparison of the thickness of central airway wall in different subtypes of TBTB patients based on EB-OCT imaging.

	Mucosa to intima of cartilage (μm) (mean ± SD)		*P*-value	Cartilage (μm) (mean ± SD)		*P*-value
Normal vs II (*n* = 64:3)	379 ± 73	505 ± 21	0.005	1,597 ± 368	1,506 ± 59	0.135
Normal vs. UN (*n* = 64:2)	379 ± 73	779 ± 1	<0.001	1,597 ± 368	1,474 ± 28	0.021
Normal vs. GH (*n* = 64:7)	379 ± 73	1,849 ± 306	<0.001	1,597 ± 368	–	–
Normal vs. CS (*n* = 64:38)	379 ± 73	687 ± 115	<0.001	1,597 ± 368	1,217 ± 430	<0.001
Normal vs. TM (*n* = 64:25)	379 ± 73	679 ± 209	<0.001	1,597 ± 368	–	–
II vs. UN (*n* = 3:2)	505 ± 21	779 ± 1	<0.001	1,506 ± 59	1,474 ± 28	0.540
II vs. GH (*n* = 3:7)	505 ± 21	1,849 ± 306	<0.001	1,506 ± 59	–	–
II vs. CS (*n* = 3:38)	505 ± 21	687 ± 115	0.001	1,506 ± 59	1,217 ± 430	0.001
II vs. TM (*n* = 3:25)	505 ± 21	679 ± 209	0.169	1,506 ± 59	–	–
UN vs. GH (*n* = 2:7)	779 ± 1	1,849 ± 306	<0.001	1,474 ± 28	–	–
UN vs. CS (*n* = 2:38)	779 ± 1	687 ± 115	0.277	1,474 ± 28	1,217 ± 430	0.410
UN vs. TM (*n* = 2:25)	779 ± 1	679 ± 209	0.512	1,474 ± 28	–	–
GH vs. CS (*n* = 7:38)	1,849 ± 306	687 ± 115	<0.001	–	1,217 ± 430	–
GH vs. TM (*n* = 7:25)	1,849 ± 306	679 ± 209	<0.001	–	–	–
CS vs. TM (*n* = 38:25)	687 ± 115	679 ± 209	0.828	1,217 ± 430	–	–

II, inflammatory infiltration; UN, ulcer necrosis; GH, granulation hyperplasia; CS, cicatricial stenosis; TM, tracheobronchial malacia; “-” means intact cartilage invisible.

## Discussion

The principle of EB-OCT is that the near-infrared light waves have different refractive indices in different tissues. After the light waves are analyzed and processed by a computer, a two-dimensional real-time image is generated (Han et al., 2005; Williamson et al., 2010). The near-infrared light band is harmless to patients, and it can scan a depth of 3 mm into human tissues. EB-OCT is easy to operate and it is non-invasive, repeatable, and radiation free (Montaudon et al., 2009; Price et al., 2018). EB-OCT is a novel technique to observe the bronchial wall and its tissue structure when performing bronchoscopy equipped with an OCT probe. The OCT probe can enter the airway (up to one to nine bronchi) through the bronchoscope working channel, and then, the reflected light from the bronchial wall can generate high-resolution cross-sectional images of the airway wall and accurately display the structure of the mucosal layer, submucosal layer, cartilage, and alveoli (Coxson et al., 2008; Peng et al., 2021). It can display the local micro-structure accurately and clearly, which is highly consistent with the pathological results (McLaughlin et al., 2010; Long et al., 2023).

At present, the understanding of cartilage destruction in TBTB patients comes from surgical resection of specimens or speculation of cartilage destruction through bronchoscopic observation of airway collapse. However, there is a lack of understanding *in vivo* about whether the tracheal cartilage of TBTB patients is damaged. In our study, we found that EB-OCT presented the normal and abnormal central airway cartilage, including tracheal, right main bronchial, right bronchus intermedius, and left main bronchial. These EB-OCT images provided us with an intuitive understanding of the changes in the cartilage in TBTB patients. From the sex and different location aspects, we measured the thickness of the airway wall of TBTB patients in their relatively normal central airway based on EB-OCT imaging. The results suggest that for most TBTB patients, EB-OCT can clearly display the structure of the tracheal wall, including the cartilage, even in the thickest part of the cartilage. Our study suggested that EB-OCT can be used to estimate airway cartilage in TBTB patients.

Furthermore, EB-OCT images displayed the cartilage damage in different subtypes of TBTB patients. The cartilage had varying degrees of damage in the subtypes of cicatricial stricture and tracheobronchial malacia, even cartilage deficiency. Our study suggests that cartilage destruction in TBTB patients may occur at an earlier stage, even during the ulcer necrosis stage. It increased our understanding of the *in vivo* state of the airway cartilage in TBTB patients. As far as we know, this is the first *in vivo* study to demonstrate central airway cartilage injury in TBTB patients. As research continues, we will accumulate more experience and achievements about EB-OCT. EB-OCT will be widely used to evaluate airway cartilage damage in TBTB patients.

Our research also has some limitations. First, we have not found cartilage in some patients; this may be due to excessive thickening of the mucosa to the cartilage intima that cannot detect cartilage. However, there is no conclusive evidence to confirm it. We just inferred this result based on EB-OCT imaging and clinical experiences. Second, the operation of EB-OCT and the recognition of the final image may vary among different doctors, which may lead to different interpretations of the examination results. We suggest that doctors conducting this examination and image interpretation should receive training, and when there are uncertain results, multiple doctors need to discuss them. Third, our EB-OCT imaging was performed *in vivo* and cannot be verified with pathological findings of cartilage destruction. Because we could not obtain the airway cartilage of TBTB patients, in the future, it is necessary to establish a TBTB animal model to study the pathological and physiological changes of the airway cartilage based on its important value in the process of airway diseases. This will increase our understanding in this area.

## Conclusion

Our study conducted EB-OCT examination of the central airway cartilage of TBTB patients for the first time, which increased our understanding of the *in vivo* state of the airway cartilage in TBTB patients. It also increased our understanding of the *in vivo* state of the airway wall in TBTB patients. EB-OCT helps to estimate the cartilage damage of the central airway in TBTB patients to some extent.

## Data availability statement

The original contributions presented in the study are included in the article/supplementary material. Further inquiries can be directed to the corresponding authors.

## Ethics statement

The studies involving humans were approved by The Research Ethics Commission of the First Affiliated Hospital of Chongqing Medical University. The studies were conducted in accordance with the local legislation and institutional requirements. The participants provided their written informed consent to participate in this study.

## Author contributions

KZ: Data curation, Writing – original draft, Writing – review & editing. BL: Data curation, Formal Analysis, Writing – review & editing. SL: Writing – original draft, Supervision. YL: Funding acquisition, Resources, Supervision, Writing – review & editing. SG: Funding acquisition, Supervision, Writing – review & editing.
